# The integration of mixed methods data to develop the quality of life – aged care consumers (QOL-ACC) instrument

**DOI:** 10.1186/s12877-021-02614-y

**Published:** 2021-12-15

**Authors:** Claire Hutchinson, Julie Ratcliffe, Jenny Cleland, Ruth Walker, Rachel Milte, Candice McBain, Megan Corlis, Victoria Cornell, Jyoti Khadka

**Affiliations:** 1 Caring Futures Institute, Flinders University, Adelaide, Australia; 2 College of Nursing and Health Sciences, Flinders University, Adelaide, Australia; 3 John Walsh Centre for Rehabilitation Research, Faculty of Medicine and Health, The University of Sydney, Sydney, Australia; 4 Australian Nursing and Midwifery Federation (SA Branch), Adelaide, Australia; 5 ECH Aged Care, Adelaide, Australia; 6 Registry of Senior Australians (ROSA), South Australian Health and Medical Research Institute, Adelaide, Australia

**Keywords:** Aged care, Economic evaluation, Measuring outcomes, Mixed methods, Older people, Quality of life

## Abstract

**Background:**

This paper describes the collection and integration of mixed methods data to facilitate the final selection of items for the Quality of Life – Aged Care Consumers (QOL-ACC) instrument. The aim of the wider project is to develop a preference-based quality of life instrument that can be used for quality assessment and economic evaluation. Older people have been involved at every stage of the development of the QOL-ACC to ensure that the final instrument captures their perspectives and preferences.

**Methods:**

Mixed methods data was collected on draft items for the QOL-ACC instrument across six key quality of life dimensions (mobility, pain management, emotional well-being, independence, social connections, and activities). Qualitative face validity data was collected from older people (aged 66 to 100 years) living in the community and in residential aged care via semi-structured interviews (*n* = 59). Quantitative data was collected from older people (aged 65 to 91 years) receiving aged care services in the community via an online survey (*n* = 313). A traffic light pictorial approach was adopted as a practical and systematic way to categorise and present data in a meaningful way that was easy for non-academic workshop members to understand and to be able to discuss the relative merits of each draft item.

**Results:**

The traffic light approach supported the involvement of consumer and aged care provider representatives in the selection of the final items. Six items were selected for the QOL-ACC instrument with one item representing each of the six dimensions.

**Conclusions:**

This methodological approach has ensured that the final instrument is psychometrically robust as well as meaningful, relevant and acceptable to aged care consumers and providers.

**Supplementary Information:**

The online version contains supplementary material available at 10.1186/s12877-021-02614-y.

## Introduction

As with many developed nations, Australia’s population is ageing [1, 2]. Currently, over one in seven Australians are older people (15%) (aged 65 years and above) [3]. This cohort of the population is expected to rise to more than 1 in 5 people (20%) by 2057 [3]. In particular, the number of people aged 85 years and above – and much more likely to be accessing residential aged care services – has more than doubled in the last two decades, placing increased demand on aged care services [1, 4]. Australia’s proportion of older people is similar to other developed nations such as the US, UK, Canada and New Zealand, and globally it is predicted that there will be 2.1 billion older people by 2050 and 3.1 billion by 2100 [5].

The aged care sector in Australia provides services in the community via the Commonwealth Home Support Program (entry level support) and Home Care Packages (from Level 1 - low care support, to Level 4 - high care support) as well as providing services via residential care homes [6, 7]. Remaining in their own home in the community is the preferred option of many older people [7–9] and use of home care has increased by 140% in the last decade [4]. Despite this, Australia remains one of the most highly institutionalised aged care systems in the world [10]. In 2018–2019, Government expenditure on the aged care sector was $20 billion, 66% of which was spent on residential aged care [11]. This represents a 27% increase in government aged care expenditure since 2013–2014 [11].

The recent Australian Royal Commission into Aged Care Quality and Safety has highlighted many failings in the system [12–14] and the need for ongoing measurement and monitoring of the quality of care provided and the overall quality of life of people receiving care. Currently quality of care is routinely measured in residential care during accreditation auditing every three years but is predominantly clinically-focused e.g. weight loss/nutrition, pressure ulcers, etc. [2, 6]. The measurement of quality of life is not currently mandated in Australia’s aged care system, though the final submission by Assisting Counsel to the Royal Commission recommends that quality of life is routinely measured by aged care providers commencing 1 July 2023 [14]. Quality of life data is currently collected as part of routine monitoring in other similar developed nations including the UK, US, Canada and New Zealand [15].

In addition to being used to measure and monitor quality of life as part of quality assessments by aged care providers, preference-based quality of life instruments can also be used for economic evaluation. Preference-based instruments consider the relative importance of different quality of life dimensions and can be used to generate quality adjusted life years (QALYs). QALYs are used as the outcome in cost-utility analysis which is the recommended form of economic evaluation for health-based interventions in the UK, Australia, and the Netherlands [16–18]. Individual responses are converted into a utility score (from 0 (worst) to 1 (best)) using a scoring algorithm. In some cases, negative utility scores can also be generated indicating quality of life states considered to be worse than being dead. Preference based scoring algorithms are typically derived from large general population samples using established valuation methods widely applied by health economists and health service researchers including time trade off (TTO), standard gamble (SG) or discrete choice experiments (DCE) [19].

Few quality of life instruments have been developed specifically to measure the quality of life of older people, or more specifically older people using aged care services [19]. The ICEpop CAPability instrument for Older people (ICE-CAP-O) was developed with older people living independently in the community in the UK and is preference-based [20]. The underlying theoretical model for the instrument is Sen’s capability theory [21] which differentiates it from other existing preference-based quality of life instruments. The ICECAP instrument is not anchored on the QALY scale and thus cannot be used to calculate QALYs for application in cost-utility analysis [22]. Though aimed at older people it was not specifically developed for or with aged care consumers.

The Adult Social Care Outcomes Toolkit (ASCOT) [23] was developed in the UK for adults of all ages in a broader social care context. The ASCOT instrument was not developed specifically for application with older people receiving aged care services and, as such, it might not best reflect the quality of life dimensions that are most important to this population. Another quality of life measure that has been used extensively for economic evaluation in health care sectors internationally and also applied in social care sectors in several countries is the EQ-5D [24] which has preference-based scoring algorithms for a three-response level version [25] and five-response level version [26]. Though widely used, this quality of life instrument was developed with adult populations rather than older people and, as with the ASCOT, may not reflect the quality of life dimensions, nor language, most meaningful and preferred by older people who use aged care services [22].

Generic adult instruments tend to focus on health and, whilst older people have been shown to value physical health as they age, there is evidence that older people also value a range of non-health domains such as independence and safety [27] which may make such instruments insufficient in isolation to capture older people’s quality of life. Indeed, in a study examining the importance of quality of life domains to older and younger people, marked differences were noted with younger people considering mental health the most important quality of life domain, and older people most valuing independence and having control over their lives [28]. Thereby indicating that physical health was not the most important quality of life domain. It is therefore important to develop a quality of life instrument which captures what older people consider to be most important for them to have a good life as they age.

Informed by this previous research, the current study is part of a wider three-year project to develop a preference-based quality of life instrument suitable for use with older people in both community and residential aged care settings; the Quality of life – Aged Care Consumer (QOL-ACC). This project has adopted a bottom-up approach from its inception, including older people in each stage of the development of the instrument [29]. That is, the instrument was developed from in-depth qualitative interviews about what quality of life means to older people receiving aged care services rather than top-down from literature reviews, systematic reviews, or expert opinion, which has been a common starting point for several quality of life instruments (e.g. [25, 30, 31]). Across the stages, the project has included older people accessing aged care, whether in the community (Commonwealth Home Support Packages and Home Care Packages), or in residential aged care [29]. Throughout we sought to be as inclusive as possible, collecting data from older people with mild to moderate cognitive impairment or dementia (where informed consent could be provided) as well as those with normal cognition.

Prior to the commencement of the project in 2018, a steering group was set up to guide the project. The members of the steering group include the research team, representatives from five aged care providers providing services across five states and territories (South Australia (SA), New South Wales (NSW), Victoria (VIC), Tasmania (TAS), Australian Capital Territory (ACT)), consumer representation and a representative from the Aged Care Quality and Safety Commission. The Aged Care Quality and Safety Commission’s (ACQSC) is a federal Government agency whose role is “to protect and enhance the safety, health, well-being and quality of life of people receiving aged care” as the regulator of the Australian aged care standards [32].

**Fig. 1:**
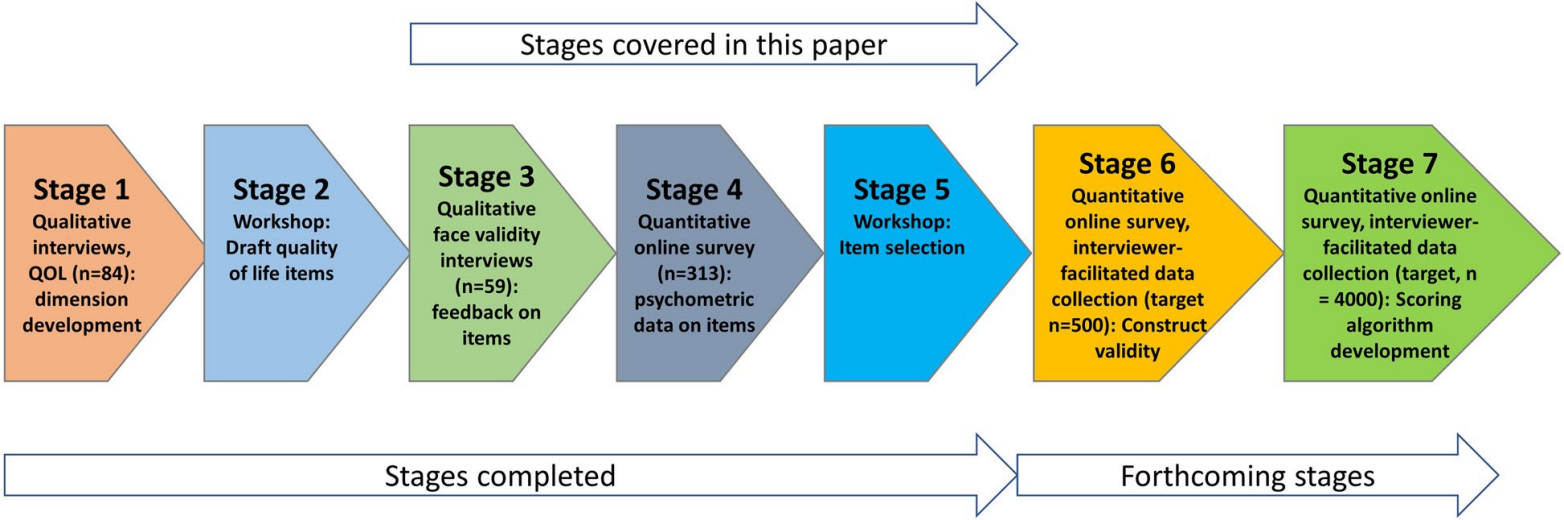
The seven stages of the development of the QOL-ACC

In stage 1 of the project, older people (*n* = 84) participated in semi-structured interviews in the community (*n* = 41) and in residential aged care (*n* = 43) (Fig. 1). These interviews identified what quality of life meant to older people and which factors most influenced their perceptions regarding their current quality of life [33]. The six dimensions that were identified as being most salient to older people’s quality of life were: mobility, pain management, emotional well-being, independence, social connections, and activities. At this stage we noted that, relative to existing quality of life instruments that included these domains, older people talked very differently about mobility and pain. For example, older people who used mobility aids still considered themselves mobile and most older people did not expect to be pain free but rather spoke of managing pain [33]. In stage 2, a workshop was held with all members of the research team, as well as consumer and aged care provider representation, to review the qualitative data and to prepare draft items for each of the six dimensions, using the language, content and terminology used by the participants themselves in describing what quality of life means to them. During this workshop 34 items were developed with frequency selected as the basis for response options, given that this is the way in which older people discussed these domains and how they related to their own life experiences. This paper details stages 3 to 5 of the project, that is, qualitative face validity interviews with older people in the community and residential aged care settings (stage 3), psychometric data on draft items from a quantitative online survey (stage 4) and the integration of these mixed methods findings to select the final items for the QOL-ACC instrument (Stage 5). Forthcoming stages are to further test construct validity (stage 6) and to develop a preference-based scoring algorithm (stage 7) (Fig. 1).

## Methods

This section outlines the qualitative and quantitative data that were collected, and how this data was integrated, with the aim of identifying the final items for the QOL-ACC instrument.

### Qualitative data

#### Participants

Participants in Cohort 1 were recruited via five aged care providers across three states (SA, NSW, TAS). To be eligible for the study, participants had to be aged 65 years and over, receiving a government Home Care Package, able to communicate in English, and have normal cognition through to mild cognitive impairment / mild dementia (as assessed by the aged care organisation using the Psychogeriatric Assessment Scale Cognition Impairment Scale (PAS-Cog) [34]. Participants in Cohort 2 were recruited via three aged care providers across two states (SA, NSW) and were receiving aged care services in residential care settings. Cohort 2 did not include participants from Tasmania as our interviewers were based in SA and NSW, and the border to Tasmania closed due to COVID-19 between the first and second round of data collection. Eligibility criteria in relation to age, cognition and ability to speak English were as described above.

Given the need to work around restrictions in various states, and that people receiving aged care services as identified as being a hard-to-reach group [35, 36] convenience sampling was adopted [37], with all older people who met the criteria and wished to participant being interviewed.

#### Materials

An interview protocol was developed for the interviewers conducting the face validity interviews (see Supplementary materials). This protocol was initially drafted by two of the authors (CH, JC) and subsequently developed and agreed with the steering group, including consumer and aged care providers representation. This included the generated items (from Stage 2) arranged by quality of life dimension, as well as prompts for the interviewer to elicit feedback e.g. How do you interpret the question? Is the item clear and understandable? Is the wording appropriate for older people? Would you be prepared to answer this question? Do you think other people would find this question acceptable? Is your preferred response available? Which of the options available do you like the best and why? Is there anything that is important to your quality of life that isn’t captured in these questions?

Cohort 1 was also presented with five response sets based on frequency to elicit their preferred response set. Based on feedback from Cohort 1, one response set (‘none of the time’ to ‘all of the time’ was presented to Cohort 2 along with the remaining items (see supplementary materials for full item bank and response set options).

A series of socio-demographic questions were included in the interview protocol to capture participants age, gender, postcode, country of birth, and highest educational qualification. For Cohort 1 (community sample) we also asked about living arrangements and home care package level. Participants were also asked to complete the EQ-5D-5L instrument assessing health-related quality of life [26].

#### Procedure

Aged care providers approached eligible participants who were provided with a Participant Information Sheet. Once participants indicated their verbal consent to participate, their names and contact details were then passed to the research team by the aged care provider so that interviews could be arranged at a convenient time for participants. Interviews took place in the participants home (Cohort 1) or in their room at their residential care facility (Cohort 2). Informed consent was obtained prior to interviews commencing and interviews were recorded with the permission of participants. Data was collected between April and June 2020.

#### Analysis

Audio recordings were transcribed by a professional transcription service under a signed confidentiality agreement with the university. Transcripts were imported into NVivo 12 and data was collated under each of the 34 items independently by two researchers (JC, CM). Coding was finalised via discussion, with a third researcher (CH) making the final decision where agreement could not be reached. Data were coded to reflect item feedback regarding the clarity, ambiguity, judgemental wording, sensitivity, acceptability and preferred wording of the draft items. The aim was that all items that were clear, unambiguous, acceptable, and used preferred wording would be carried forward for psychometric testing, with no targets set for item reduction at this stage.

### Quantitative data

#### Participants

Participants were recruited via an online panel company. To be eligible participants had to be 65 years and over, receiving either a Commonwealth Home Support Program (CHSP) or a Home Care Package. Approximately 840,000 older Australians access their aged care services via CHSP [11], and 145,000 via Home Care Packages [38]. However, whilst the intention of the CHSP program is that it provides services to older people with lower levels of care needs, in practice there is currently a large waiting list for the greater level of care provided by the HCP program [39], so there is a lot of overlap between the clients for these two programs. Face to face interviewer-facilitated data collection in residential care facilities was not possible due to COVID-19 restrictions in various states.

#### Materials

In section A of the online survey, participants were presented with the draft QOL-ACC items with five response options: ‘none of the time’, ‘a little of the time’, ‘some of the time’, ‘most of the time’ and ‘all of the time’. Section B was the six item Quality of Care Experience (QCE) questionnaire [40], an instrument derived from the quality in aged care standards and an expert panel in health, ageing and health economics as part of a Royal Commission project [7]. Section C presented participants with the Adult Social Care Outcomes Toolkit (ASCOT) [23]. Section D consisted of the EQ-5D-5L [26]. Section E consisted of the Personal Wellbeing Index Scale [41], a cross-cultural measure of subjective well-being. The survey opened in April 2021 and data was collected for a period of six weeks.

Prior to analysis, the online panel company conducted a number of data quality checks. Respondents who complete the survey too quickly (‘speedsters’) were removed as were ‘flatliners’ (those who selected the same response option throughout the survey or a whole section). The responses of people who took longer than average to complete the survey were reviewed for inattentiveness. Duplicates were removed if a person had completed the survey more than once (based on IP address).

#### Analysis

The raw responses to the QOL-ACC draft items across 6 dimensions were subjected to Classical Test Theory (CTT) and modern psychometric methods (Rasch model analysis). The CTT and Rasch are the best practice methods of assessing the psychometric properties and validity of a new instrument [42–45]. When combined, CTT and Rasch analysis metrics provide better insights into important psychometrics properties both at an instrument and item level. In the study, a series of CTT and Rasch analysis based psychometric parameters for items within each dimension were assessed and compared, with an aim to identify a single high quality, informative and psychometrically robust item per dimension. STATA version 15.0 was used to conduct descriptive statistical analysis as well as to investigate psychometric properties related to Classical Test Theory. Winsteps version 4.7.1.0 was used to conduct the Rasch analysis. To assess psychometric properties at item level, the Masters Partial Credit Model (PCM) for Rasch analysis was used, whereby the response structure is modelled to be unique to each item independent of response structure of other items. The PCM is a non-restrictive Rasch model which allows each item to have its own independent set of response category structure. That is PCM does not impose a common thresholds structure across all items allowing each item to be defined as own partial credit scale [46]. As the final QOL-ACC descriptive system has 6 domains defined by a single item, the PCM was deemed appropriate as it allowed all 6-items to behave independently within the model.

The CTT based statistics include evaluation of acceptability, targeting, internal consistency reliability (Cronbach’s alpha) and item dependency. The Rasch analysis statistics include category function test, item fit statistics, test information function and differential item functioning by sex, age group and service type.

### Data integration: the ‘traffic light’ system

**Fig. 2:**
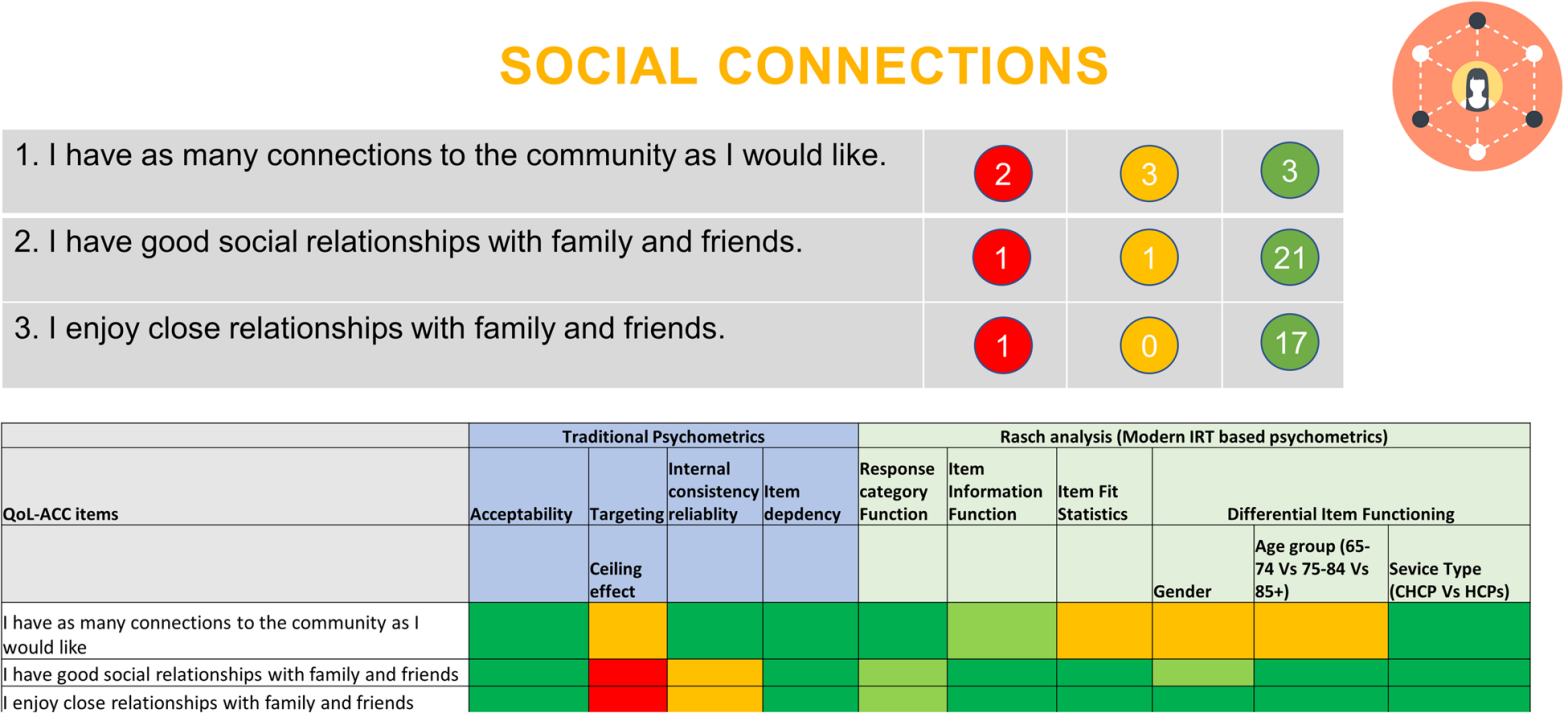
Example of Traffic Light coding for the QOL-ACC dimension of social connections

A workshop was attended by an expert panel including the research team, aged care provider and consumer representatives. Prior to the workshop the data from the qualitative and quantitative phases was coded as red, amber and green according to predefined criteria and presented on summary slides where the coding for all criteria could be viewed at once for the potential items for a dimension (see Fig. 2). In this way, we followed similar methodology to that of Keetharuth et al. 2018. [30] in the development of the ReQoL, a measure of quality of life for those recovering from mental health conditions, which was influenced by the work of Luyt [47] and Adcock and Collier [48].

For the qualitative data, feedback from older people regarding ambiguous wording, judgemental wording, potentially sensitive or distressing wording were coded as red. Feedback that items were clearly worded, unambiguous and represented preferred wording were coded as green. Any mixed or neutral comments were coded as amber. Rather than coding each item as green, amber or red overall as Keetharuth et al. did [30], the number of quotes coded as red, amber and green from the face validity interviews were retained (see coloured circles on Fig. 2 as an example).

For the quantitative data, a comprehensive set of psychometric properties were assessed and graded for each item based on a predetermined quality criteria [49, 50] (Table 1). The psychometric properties and the quality criteria align with the best practice guidelines proposed by the Consensus-based Standards for the selection of health status measurements (COSMIN) and the U.S. Food and Drug Administration (FDA) [42, 44]. These guidelines provide best practice checklist in evaluating quality of the patient-reported outcome instruments used to determine end points in outcome studies and clinical trials. For the ease of interpretation, the psychometric properties for each item were graded as excellent/very good (‘A’ – coded as ‘green’), acceptable (‘B’ - coded as ‘amber’) and poor psychometric properties (‘C’ - coded as ‘red’) (Table 1). An example of the qualitative and quantitative traffic light data presented to the expert panel at the workshop is shown in Fig. 2.

**Table 1:** Description of the traffic light system based on psychometric proprieties (A = Green, B = Amber and C = Red)

Classical Test Theory Based psychometric properties	
Parameters	Grading/Description
Acceptability	A: The percentage of missing data: ≤ 5% B: The percentage of missing data: > 5% ≤ 40% C: The percentage of missing data: > 40%
Targeting	A: End-point categories ≤5% B: End-point categories > 5% ≤ 40% C: End-point categories > 40%
Internal consistency reliability	A: 0.95 ≥ Cronbach’s α ≥ 0.70 B: 0.70 > Cronbach’s α ≥ 0.60 or Cronbach’s α > 0.95 C: Cronbach’s α ≥ 0.60
Item dependency	A: Inter-item correlations < 0.3 B: Inter-item correlations ≥0.3 < 0.6 C: Inter-item correlations ≥0.6
**Modern Psychometrics (Rasch Analysis) based properties**	
Response categories	A: All the categories are ordered and evenly spaced B: All the categories are ordered but categories are nor evenly spaced C: Disordered categories
Item fit statistics	A: Item with infit and outfit mean square (MNSQ): 0.70–1.30 B: Item fit statistics outside 0.70–1.30 limit but within 0.5–1.5 limit C: Item fit statistics outside 0.5–1.5 limit
Differential Item Functioning (DIF) (assessed for gender and service type)	*DIF tests whether population subgroups respond to an item differently. It is assessed by examining the mean difference in item measure between the groups. DIF was assessed for gender (male* vs *female), age groups (65–74 yrs* vs *75–84 yrs* vs *85 yrs+) and service (Commonwealth Home Support Program* vs *Home Care Packages).* A: DIF ≤ 0.5 logits B: DIF > 0.5 ≤ 1.00 logits C: DIF > 1.0 logits
Item Information Function (IIF)	*IFF is the amend of information an item possesses. It is observed in a graph which is plotted between item information level (range from 0 to 1) on the Y-axis and relative item measure in logits on the X-axis.* A: Item with high-level information and wider measurement range (a bell-shaped graph) B: Item with low-level information and wider measurement range/item with high information and narrow measurement range C: Item with a low-level information and narrow measurement range

The format of the session and the aims were outlined and agreed upon at workshop commencement. An overview of the sample sizes and demographics were presented. The analytical process followed for analysing the qualitative and quantitative data was described as well as how the data was coded using the ‘traffic light’ system. The workshop members had access to the detailed psychometric analysis as well as the face validity data during the workshop. The traffic light data was presented for each of the six dimensions in turn. Where an item was the most preferred item based on face validity data, and the strongest psychometrically, this item was selected. Where the qualitative and quantitative evidence was more mixed, the strongest items were discussed between the workshop members with the aim of achieving consensus on the selection of the final item for each domain.

## Results

### Qualitative data

This data was collected from two cohorts. Cohort 1 consisted of older people receiving aged care services in the community (Home Care Packages) and Cohort 2 of older people accessing services in residential aged care. Cohort 1 consisted of *n* = 31 older people (age range 66 to 95 years, mean = 80.4 years). Cohort 2 consisted of *n* = 28 older people (age range 74 to 100 years, mean = 84.4 years) (Table 2). As expected, the residential care cohort (Cohort 2) was generally older than the community cohort (Cohort 1), with 21.4% over the age of 90 years of age, compared to only 9.7% from the community. Cohort 2 also contained more males (46.4% versus 32.3% in the community sample) and were more likely to be Australian born and with lower education levels (Table 2). When compared to national data on older people accessing home care packages and residential care, the community cohort included a higher proportion of older people (> 90 years) and people on higher level care packages (Levels 3 and 4). The residential care sample disproportionately sampled women (Table 2). Therefore, the samples included a broad cross section of older people by age, gender, and home care package (in the case of the community sample) but was not fully representative of either the home care or residential care population Australia-wide.

**Table 2:** Demographics – Face validity interviews

	Community (HCP) Sample (Cohort 1) N (%)	Community (HCP) Australia- wide (%)^b^	Residential Care Sample (Cohort 2) N (%)	Residential Care Australia-wide (%)^b^
Gender				
Male	10 (32.3)	36	13 (46.4)	37.3
Female	21 (67.7)	64	15 (53.6)	62.7
Age				
65–79 years	12 (38.7)	35	8 (28.6)	26.6
80–89 years	16 (51.6)	47	14 (50.0)	52.1
> 90 years	3 (9.7)	18	6 (21.4)	21.4
Country of birth				
Australia	22 (71.0)		24 (85.7)	
UK	6 (19.4)		3 (10.7)	
Other Highest educational level	3 (6.6)		1 (3.5)	
No qualifications	5 (16.1)		5 (17.9)	
Completed high school	3 (9.7)		14 (50.0)	
Undergraduate degree/professional qualification	10 (32.3)		5 (17.9)	
Postgraduate qualification	3 (9.7)		–	
Other	10 (32.3)		4 (14.3)	
Home Care Package Level				
Level 1 (basic care needs)	1 (3.2)	9	–	
Level 2 (low care needs)	16 (51.6)	44	–	
Level 3 (intermediate care needs)	4 (12.9)	19	–	
Level 4 (high care needs)	9 (29.0)	28	–	
EQ-5D-5L Score, mean (SD)^a^	0.63 (0.18)		0.68 (0.21)	
EQ-VAS Score, mean (SD)	59.10 (22.22)		73.39 (17.64)	

^a^Van Hout et al. [51], ^b^Khadka et al. [52]

Cohort 1 were presented with the 34 items generated at Stage 2 (draft quality of life item development). Following analysis of the face validity data from Cohort 1, 16 items were deleted as being unclear, ambiguous, containing sensitive wording, asking more than one question or being least preferred items. Some examples of the deleted items are shown in Table 3.

**Table 3:** Examples of deleted items following Cohort 1 analysis

Item	Reason	Illustrative quote
I live the life I choose and make my own decisions (Independence)	Unclear, 2 questions	*It’s like two questions in one...they are 2 separate questions…Well sometimes to live the life you choose is not up to you to make decisions about that life, its other things and circumstances.* (Participant 3, Male, 76 years).
I am physically mobile (Mobility)	Unclear	*….which suggests to me that you don’t have to use walking aids.* (Participant 4, Female, 74 years). *If you made six something like, I’m physically mobile with an aide. That extends that question a little bit, doesn’t it?* (Participant 9, female, 85 years)
I am mobile (Mobility)	Unclear, lack of qualifiers	*I’m physically mobile but not to any distance* (Participant 22, Male, 85 years)
I am able to get around as much as I need to (Mobility)	Ambiguous	*Some people find the distinction between wants and needs a bit of a problem* (Participant 7, female, 80 years)
I have enough leisure activities / hobbies to keep me busy (Activities)	Superfluous words	*Maybe the word ‘busy’ may not need to be there because if they’re enjoying them, they’re obviously spending time on them…they’re probably busy, you know.* (Participant 29, Female, 74 years).
I feel happy and free from worry (Emotional Well-being)	Sensitivity	*See ‘worry’ to me is a word I don’t even like. I don’t even use it. I don’t really like to use ‘stress’ [wording from another deleted item] too often as well.* (Participant 19, Male, 80 years).
I am free from worry and stress (Emotional Well-being)	Sensitivity, ambiguous	*It seems to be a mixture that question* (Participant 22, male, 85 years)

Cohort 1 (home care participants) were presented with five possible response categories, in addition to the generated items, to identify a preferred response set. All response sets were based on frequency, that is, the frequency with which the participant experiences each quality of life dimension. Cohort 1’s most preferred response set options was the five-level scale ‘none of the time’, ‘a little of the time’, ‘some of the time’, ‘most of the time’ and ‘all of the time’. Cohort 2 was therefore presented with the remaining 18 items and this preferred five-level response set.

### Psychometric assessment

Out of 1878 people initially approached by the online panel company, 313 met the eligibility criteria and completed the online survey. Overall, 54.6% of the sample was female and 76.4% were Australian born. Participants ranged in age from 65 to 91 years (mean 74.57 years), though predominantly were younger than those who were interviewed, with 81.2% being in the youngest age category (65 to 79 years). Participants were from six states and territories and 41.9% reported living alone. Only 14.7% described their overall health as excellent or very good (Table 4). Deloitte Access Economics [53], our sample was not representative of older Australians using community care packages, particularly under-sampling people receiving Commonwealth Home Support Program and over-sampling HCP 1 and 2 recipients. However, the largest proportion of respondents were in receipt of CHSP (Table 4).

**Table 4:** Online survey sample demographics

	Survey sample N (%)	National data N (%)^b^
Gender		
Male	142 (45.4)	
Female	171 (54.6)	
Age		
65–79 years	254 (81.2)	
80–89 years	56 (17.9)	
> 90 years	3 (1.0)	
Community care package:		
Commonwealth Home Support Program (basic care needs)	120 (38.3)	840,984 (87.3)
Home Care Package Level 1 (basic care needs)	58 (18.5)	1145 (0.1)
Home Care Package Level 2 (low care needs)	68 (21.7)	43,080 (3.7)
Home Care Package Level 3 (intermediate care needs)	25 (8.0)	43,080 (4.5)
Home Care Package Level 4 (high care needs)	27 (8.6)	43,041 (4.5)
Unsure	15 (4.8)	
Country of Birth		
Australia	238 (76.4)	
UK	33 (10.5)	
Other	42 (13.4)	
State/Territory		
ACT	4 (1.3)	
NSW	88 (28.1)	
NT	0 (0)	
QLD	95 (30.4)	
SA	29 (9.3)	
TAS	0 (0)	
VIC	72 (23.0)	
WA	25 (8.0)	
Highest educational level		
No qualifications	42 (13.4)	
Completed high school	95 (30.4)	
Undergraduate degree/professional qual	109 (34.8)	
Postgraduate qualification	44 (14.1)	
Other	23 (0.3)	
Living arrangements		
Living alone	131 (41.9)	
Living with spouse/partner	158 (50.5)	
Living with other relatives	16 (5.1)	
Living with others – not relatives	8 (2.6)	
Self-reported health		
Excellent	1 (0.3)	
Very good	45 (14.4)	
Good	104 (33.2)	
Fair	121 (38.7)	
Poor	42 (13.4)	
EQ-5D-5L Score, mean (SD)^a^	0.67 (0.23)	
EQ-VAS Score, mean (SD)	63.4 (21.02)	

^a^ Van Hout et al. [51], ^b^Deloitte Access Economics [53]

**Fig. 3:**
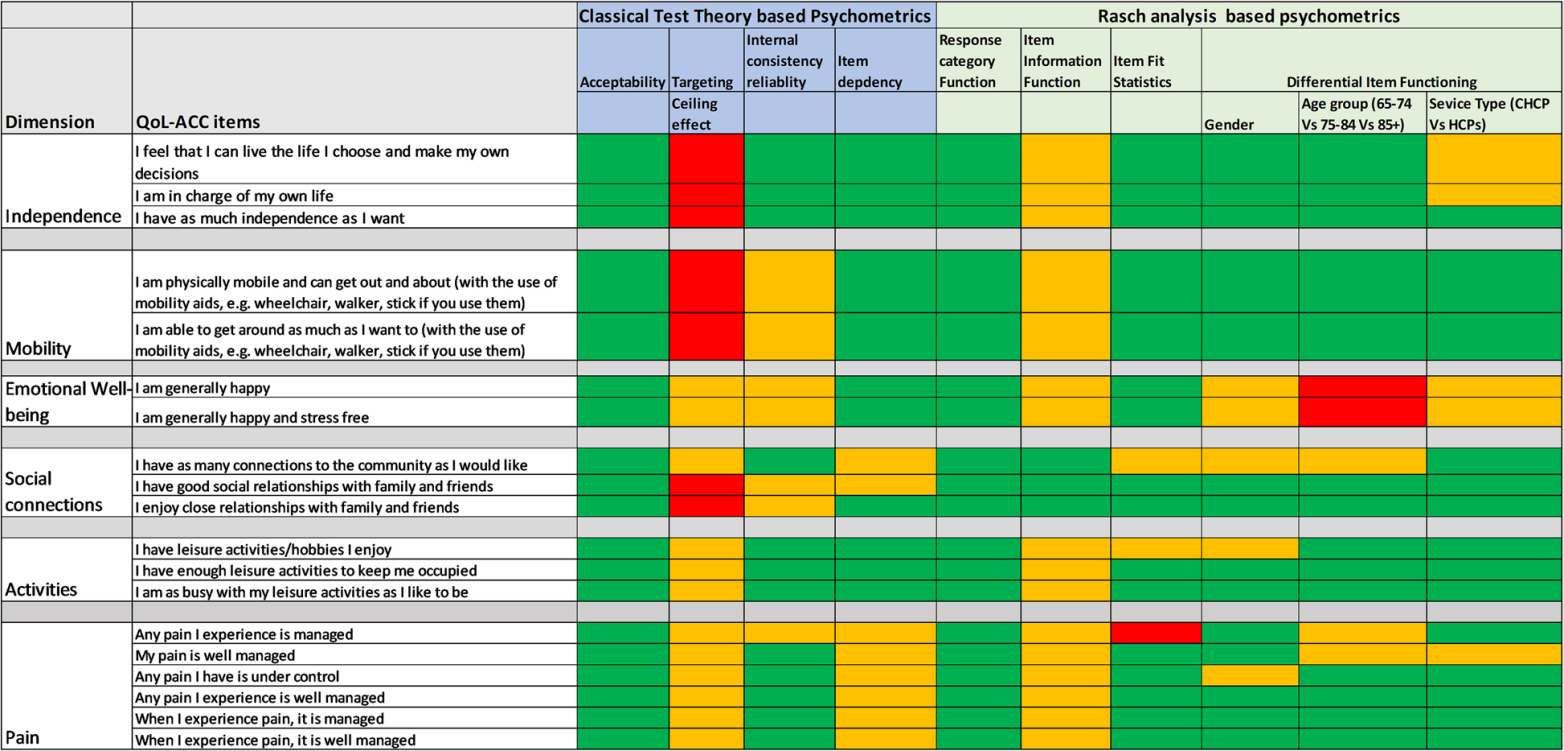
Traffic light coding of the QoL-ACC draft items based on their psychometric properties

Based on the predefined psychometric criteria items were coded into green, amber and red (Fig. 3). None of the items had > 5% missing data, few items demonstrated ceiling effect. The response categories of all the items were utilised well, evidenced by the green coding for all items for the Response Category Function column. Most of the items fitted well within each dimension, as evidenced by the predominantly green coding for Item Fit Statistics. Except for the items in “Emotional Well-being” domain, other items demonstrated an acceptable level of DIF (Fig. 3).

### Final item selection

Based on the collated traffic light pictograph data, a panel of experts reviewed the quantitative and qualitative data for each domain. Where the most preferred item based on face validity was also the strongest (or equal strong) psychometrically, that item was selected. Where this was not the case, the expert panel reviewed and discussed the available data with a view to reaching consensus on the final item selected for that domain. On this basis, three of the final items were selected based purely on a review of the data, and three based on data and discussion (Table 5). Overall, the panel demonstrated a preference for simple wording where possible. It was preferred that one item represent each dimension where possible for ease of administration, practicality and following the convention adopted by other preference-based quality of life instruments [20, 25, 54, 55]. The members agreed that no additional secondary items were required to represent any of the dimensions, resulting in six final items as detailed in Table 5.

**Table 5:** Final QOL-ACC Items by dimension

Dimension	Item	Qualitative / Quantitative data summary
Mobility	I am able to get around as much as I want to (with the use of mobility aids e.g. wheelchair, walker, stick if you use them).	Most preferred item based on face validity data. Psychometric data was equally good for all mobility items; therefore, the most preferred item was selected.
Pain Management	When I experience pain, it is well managed.	Most preferred item based on face validity data. Psychometric data was equally good for all pain management items; therefore, the most preferred item was selected.
Emotional Well-being	I am generally happy.	2nd most preferred item based on face validity data. Psychometric data was equally good for all emotional well-being items. Workshop members preferred the simple wording of this item (the most preferred item was “I am generally happy and stress free”).
Independence	I have as much independence as I want.	2nd preferred item based on face validity data, strongest item based on psychometrics and Rasch analysis. The workshop members preferred the simple wording of this item (the most preferred item was “I feel that I can live the life I choose and make my own decisions”).
Social Connections	I have good social relationship with family and friends.	Most preferred item based on face validity data. Psychometrics data was strongest for this item and the 2nd most preferred item so most preferred item was selected by workshop members.
Activities	I have leisure activities / hobbies I enjoy.	Most preferred item based on face validity data. This item was slightly weaker on Item Fit Statistics than the other activities items. However, the workshop members prefer the simple wording of this item (compared to ‘I have enough leisure activities to keep me occupied’ which was 2nd preferred and slightly stronger based on Rasch analysis).

## Discussion

This paper described the collection and integration of mixed methods data to facilitate the final selection of items for the QOL-ACC instrument. To date, the QOL-ACC instrument has been developed from the combined perspectives of over 450 older people receiving aged care services in Australia with data gathered from metropolitan and rural communities across six Australian states and territories. The instrument reflects the most salient quality of life dimensions for older people accessing aged care services, whether in the community or in residential aged care facilities. The QOL-ACC also reflects quality of life dimensions that can reasonably be expected to be impacted by the services providers, which is important for the future use of this instrument for the economic evaluation of aged care services.

The development of the dimensions and the items themselves aimed to reflect the perspectives of older people and to retain the language they use in describing the aspects of quality of life that are most relevant to them. As such, for example, the mobility item asks QOL-ACC respondents to consider the item in the context of any mobility aids they use e.g. walking stick or walking frame. This is because our Stage 1 in-depth qualitative interviews identified that older people who regularly use these devices considered themselves to be mobile [33]. This differentiates the QOL-ACC from other quality of life instruments developed with general adult populations, where a person using a mobility device could receive the lowest mobility score if they are not prompted to consider mobility aids when making their response.

Mixed methodological approaches have previously been used in instrument and scale development for other preference based instruments including the ICECAP [20] (previously described) and the Child Health Unity Instrument (CHU-9D) which was developed from the ground up with children using a similar approach to the current study [56]. Our work was guided in principle by the work of Keetharuth and colleagues [30] who used an integrative mixed methods approach to develop their mental health related quality of life instrument based on a traffic light pictorial approach. The adoption of a similar approach resulted in data being categorised in a meaningful way that was accessible for those workshop members who were not expert in psychometric analysis to understand and to be able to discuss the relative merits of each draft item. This approach facilitated a process of item selection which is inclusive of a wide range of perspectives.

This research addresses a significant gap by developing the final descriptive system for a new preference-based quality of life instrument for application in aged care that is based upon the perspectives and preferences of older people themselves. As such, we anticipate that the final instrument will be relevant, meaningful, and acceptable to users. Future stages of the project, to test the construct validity and to develop the preference-based scoring algorithm (Stages 6 and 7), will also draw on the involvement of older people using a range of aged care services across several states, including home based and residential care in metropolitan and rural areas. Until the algorithm is available, the QOL-ACC is being piloted in several aged care organisations with a simple summative score.

### Strengths and weaknesses

A major strength of this research is that it has placed older people at the front and centre of every stage of instrument development. Participants were from several states, metropolitan and rural areas, and across a wide age range and were broadly representative of the Australian population currently using aged care services [52]. The research sought to be as inclusive as possible and qualitative data collection included older people with mild to moderate cognitive impairment or dementia (where informed consent from the person themselves could be provided) as well as those with normal cognition. This was important given that it is estimated that 53% of people in residential aged care, and 17% of those receiving community aged care, have some degree of cognitive impairment [57]. It is estimated that approximately 25% of older people in Australia are from Culturally and Linguistically Diverse (CALD) backgrounds [58]. Unfortunately, due to limited resources we were not able to facilitate the involvement of participants who were not able to communicate well in English. However, we are currently undertaking a parallel research project to the one presented here to determine the relevance and acceptability of the QOL-ACC to older people from a range of CALD communities.

The stages of the development of the QOL-ACC that are reported in this paper were conducted during the COVID-19 global pandemic. Restrictions in several states impacted upon the collection of face validity data. The overall face validity sample was smaller than the original aim of *n* = 80 (*n* = 59 interviews were conducted) and some of the later interviews had to be conducted via telephone or video conferencing. However, we observed strong patterns of preferences and similar feedback regarding many of the items (as shown in Fig. 2), indicating that data saturation had been achieved. The COVID-19 restrictions also necessitated the collection of Stage 4 psychometric data online from older people in the community only. It was the intention to conduct face to face interviewer-facilitated data collection in residential aged care facilities but access to residential care facilitates was severely restricted throughout the period in which we were collecting data, and even when restrictions eased, providers were reluctant to engage in discussion about access given changing service priorities. However, the foundational qualitative interviews to identify the quality of life dimensions and the face validity of the draft QOL-ACC items did include older people in residential care across several states and aged care providers. Additional work is planned for 2021 to further test construct validity of the instrument with participants in residential care.

## Conclusion

A traffic light pictorial approach was a practical and systematic approach to categorise and present qualitative and quantitative data in a meaningful way that supported aged care provider and consumer representation to select the final items for the QOL-ACC. This methodological approach ensured that the final instrument is psychometrically robust as well as meaningful, relevant and acceptable to aged care consumers and providers. The QOL-ACC items were developed from qualitative interviews with older people receiving aged care services about what was important for them to feel like they had a good quality of life [33] and are therefore based on what is most relevant and meaningful to older people themselves. The face validity interviews removed items that older people saw as ambiguous or potentially sensitive, and the low levels of missing data suggest that items were acceptable to participants. Our online survey provided evidence that items were psychometrically robust individually. The psychometric properties and construct validity of the final six item QOL-ACC will be tested shortly.

This research addresses a significant gap by developing a quality of life instrument ‘from the ground up’ with older people using aged care services. This critical work commenced in 2018 and is timely given that the Royal Commission into Aged Care Quality and Safety has now recommended that quality of life data be routinely collected by aged care providers in Australia from mid-2023 [14]. The final stages of the project to test the construct validity of the instrument (stage 6) and to develop a scoring algorithm (stage 7) will take place in 2021. Additional work to test the acceptability and relevance of the instrument to CALD communities is underway, as well as work to produce an interviewer-facilitated self-complete version and a proxy version. Though developed in the Australian context, we anticipate that the instrument will also have international applicability, where it may be used as a standalone measure of quality of life or in combination with other instruments, including condition specific quality of life measures, for older populations.

## Supplementary Information

**Additional file 1.**

## Data Availability

The datasets used and/or analysed during the current study are available from the corresponding author on reasonable request.
