# Supplementary material for: The integration of mixed methods data to develop the quality of life – aged care consumers (QOL-ACC) instrument
Source: BMC Geriatr. 2021 Dec 15;21:702. doi: 10.1186/s12877-021-02614-y (PMC8672336; doi:10.1186/s12877-021-02614-y)
Supplement: Supplementary file 1 — Additional file 1. [file 12877_2021_2614_MOESM1_ESM.docx]

**Supplementary materials**

1. Interview guide

Developing a new Quality of Life instrument for older people

INTERVIEWER GUIDE

1. **Introduction**

We are working on a project to develop a new quality of life instrument with older people. This instrument will be used for quality assessments and for economic evaluations.

We have already interviewed over 80 people in residential aged care and in the community to ask them about what having a good quality of life means to them.

From these interviews we have identified six areas that are the most important to the older people we have spoken to. We have now developed some draft questions for each of these areas and are now talking to people to find out what they think of these draft questions and response options.

First, I need to get your formal consent to participate in the research. Then I will go through the draft questions (and response options) with you. At the end I will ask you a few basic questions about you, the services you received and your health.

1. **Consent Process**

***Go through consent form with participant and answer any questions they have. If they do not provide consent, end the session.***

Are you ready to get started? [comfortable, need glasses?]

***[Start audio recording]***

1. **Draft items and response categories**

I am going to go through 6 sets of questions (and response options) with you. We are looking for feedback on whether the items are clear and understandable, appropriately worded and whether your preferred answer is available.

Let’s get started. Here is the first set of draft questions and responses….***[go through all sets]***

***Let the participants give their initial thoughts unprompted. Based on what they say, you can use the following prompts as appropriate:***

- How do you interpret the question?
- Is the item clear and understandable?
- Is the wording appropriate for older people?
- Would you be prepared to answer this question?
- Do they think other people with understand the question / find it acceptable?
- Is your preferred response available? If not, what would your preferred response be?
- What do you think about the definitions? Do they help? Are they clear?
- Which of the options available do you like the best and why?
- Is there anything that is important to your quality of life that isn’t capture in these questions?
- Are there any questions that you don’t think are important to your quality of life?

1. **Socio demographic questionnaire & EQ-5D**

Thank you for participating. We appreciate your feedback and help with refining the questions for the quality of life measure.

1. Item Bank

| Domain | Draft items |
| --- | --- |
| Independence | 1. I live the life I choose and make my own decisions 2. I feel that I can live the life I choose and make my own decisions 3. I am in charge of my own life 4. I have as much independence as I want |
| Mobility | 1. I am mobile 2. I am able to get around as much as I need to 3. I am able to get around as much as I want to 4. I find it easy to move around 5. I am able to move around 6. I am physically mobile 7. I am physically mobile and can get out and about |
| Emotional Well-being | 1. I am happy and free from worry 2. I am generally stress free / free from stress 3. I am generally happy 4. I am free from worry (and stress) 5. I am generally happy and stress free |
| Social Connections | 1. I have as many social connections as I need 2. I have as many social connections as I would like 3. I have good social relationships with family and friends 4. I enjoy close relationships with family and friends 5. I am happy with my close friendships 6. I have as much contact as I like with family and friends |
| Activities | 1. I have enough (leisure) activities / hobbies to keep me busy 2. I am as busy as I would wish with my leisure activities 3. I am as busy with my leisure activities as I like to be 4. I feel that I have enough leisure activities to keep me busy 5. I feel that I have enough leisure activities to keep me occupied 6. I have leisure activities / hobbies I enjoy |
| Pain Management | 1. Any pain I experience is managed 2. My pain is well managed 3. Any pain I have is under control 4. Any pain I experience is well managed 5. When I experience pain, it is managed appropriately 6. When I experience pain, it is well managed |
